# Supplementary material for: The Role of Endoscopy in the Palliation of Pancreatico-Biliary Cancers: Biliary Drainage, Management of Gastrointestinal Obstruction, and Role in Relief of Oncologic Pain
Source: Cancers (Basel). 2023 Nov 10;15(22):5367. doi: 10.3390/cancers15225367 (PMC10670525; doi:10.3390/cancers15225367)
Supplement: Supplementary file 1 [file cancers-15-05367-s001.zip › cancers-2635184-SI.pdf]

Supplementary Materials

Materials and Methods

The search strategy included comprehensive search in the main international databases as PubMed, Medline and Scopus regarding endoscopic palliation of bilio-pancreatic malignancies in order to provide a complete and detailed overview of the topics. Since the argument explores many different clinical scenarios of advanced bilio-pancreatic cancers, the search strategy was applied and adapted for each topic (Malignant biliary obstruction [MBO], malignant gastric outlet obstruction [MGOO] and intractable oncologic pain treated by endoscopic procedures as celiac plexus neurolysis [CPN]) using topic-related keywords. The PICO model [1] was not used to generate the search questions due to its unproven efficacy in the field of upper GI and pancreatic diseases for searching into databases such as Embase and PubMed. [2] The three main topics were explored by a search using combinations of keywords as "MBO", "malignant biliary obstruction", "malignant gastric outlet obstruction", "celiac plexus neurolysis", "MGOO", "CPN", "EUS", "endoscopic ultrasound", "endoscopy", "palliation", "biliary", "pancreatic", "cancer", "obstruction", "pain", "jaundice". Search was conducted in the main international database as PubMed, Embase, Medline and Scopus until August 2023.

Search strategy

In general, studies were included in the review if they met the following criteria: (1) they included patients with advanced bilio-pancreatic cancers; (2) they included endoscopic treatments for palliation of the malignant condition; (3) they included procedural and clinical outcomes, as clinical success and technical success, or more specific outcome procedure-related; (4) they were available as full-text publication in English language. Studies were excluded if: (1) they included patients with unclear malignant status (2) they were case reports or case series including conditions better explored in larger studies already included in the review (3) they were abstracts or interim analysis of ongoing studies, even if presented at international courses or congresses. The comprehensive search included reviews, meta-analyses, clinical trials and observational studies. To identify additional studies, a manual search of the reference lists of reviews and studies was evaluated. If any studies presented discordances with the aim of this review, three authors (G.E.M.R., L.C. and G.R.) assessed the eligibility of the studies by discussion. Data from selected studies were evaluated for relevance in relation to the outcomes of interest during the writing of the sections, and were consecutively added and gathered into sections and sub-sections.

Supplementary table S1. Bismuth Classification of Klatskin tumors [3].

**Table 1. Bismuth Classification of Klatskin tumors [3]**		
**Type I**		involving the common hepatic duct below the confluence
**Type II**		Involving the biliary confluence
**Type III**	**III A**	involving the confluence and extending to the right hepatic duct
	**III B**	involving the confluence and extending to the left hepatic duct
**Type IV**		Involving the confluence and extending to both the right and left hepatic bile duct

References

1. Richardson, W.S., M.C. Wilson, J. Nishikawa, and R.S. Hayward, The well-built clinical question: a key to evidence-based decisions.ACP J Club, 1995. 123(3): p. A12-3.

2. Frandsen, T.F., M.F. Bruun Nielsen, C.L. Lindhardt, and M.B. Eriksen, Using the full PICO model as a search tool for systematic reviews resulted in lower recall for some PICO elements. J Clin Epidemiol, 2020. 127: p. 69-75.

3. Bismuth, H., R. Nakache, and T. Diamond, Management strategies in resection for hilar cholangiocarcinoma. Ann Surg, 1992. 215(1): p. 31-8.
